# Application, ecotoxicology and regulation of insecticides with special reference to neonicotinoids

**DOI:** 10.1080/19336934.2026.2677279

**Published:** 2026-05-26

**Authors:** David Hunter, Robert Black

**Affiliations:** a Locust and Grasshopper Control, Australia; b Independent Legislative Counsel (Biosecurity/Pesticides), London, UK

**Keywords:** Pesticides, insecticides, active ingredients, plant protection products, ecotoxicology, registration, neonicotinoids, international trade

## Abstract

This review examines the efficacy and toxicity of the major insecticide groups in current use, with particular reference to neonicotinoids, which are one of the most widely used pesticides in the world. Mammalian toxicity and hazards posed during insecticide application will be discussed, along with their ecotoxicology, effects on non-target organisms and on the environment. However, toxicity must be balanced by usefulness in crop protection, first in terms of integrated pest management (IPM) and then by detailing national registration processes according to international normative frameworks to achieve the appropriate balance between efficacy and toxicity. Critical in ensuring that this balance is met are controls on pesticides in use, enforcement of pesticide regulations, and food safety measures related insecticide residues and maximum residue limit (MRL) setting. International trade conventions and trade issues are of direct relevance to import and export not only of agricultural products protected by the use of pesticides, but also of the pesticides themselves along with the waste products from their manufacture or use.

## Introduction

Pesticides and particularly insecticides in the 21st century present a double-edged sword in that the benefits in controlling pests and contributing to higher yields in crops need to be balanced with potential and actual dangers to humans, wildlife and the environment. The hazards presented by many of the pesticides in use at the time were exposed in *Silent Spring* by Rachel [1] and reinforced in *Since Silent Spring* [2]. Carson faced very fierce hostility both personally and professionally from the agrochemicals industry, but today efficacy is balanced with safety as the two foundations of pesticide registration and regulation of use. Chemical pesticides have proven valuable in arable and horticultural crop production, food storage, forestry and public health (especially disease vector control), but their side effects feature in three international conventions aimed at prevention of chemical harm to the environment.

At the time of *Silent Spring* the insecticides in use were mostly chlorinated hydrocarbons including DDT, aldrin, dieldrin, chlordane, and heptachlor. These were known for their persistence in the environment and their ability to accumulate in living organisms, while aldrin and dieldrin were extremely toxic to mammals and so were hazards to human health both during application and when in the environment. These effects led severe limitations and then outright banning of DDT during the 1970s (USA and many European countries) and 1980s (Australia, Canada, China, Japan) [3]. Not only DDT, but virtually all chlorinated hydrocarbons have now been banned in most countries.

With the banning of chlorinated hydrocarbons, other groups of chemical pesticides with shorter residual periods and lower classes of hazard came into use. This review will summarize how these chemical pesticide groups have been used, but with the increased understanding of the both the short and long-term effects of even less harmful pesticides, there has been increased scrutiny of the effects of these replacements, leading to some having severe restrictions placed on their use or even being banned altogether. Consequently, the review will outline how government agencies and registration authorities assess potential pesticides to ensure their continued use in as safe a way as possible as part of minimizing the side effects that have led to increased scrutiny and restrictions. The procedural bases for national registration systems are provided in the international normative frameworks (‘standards’) described below. It is well understood that control of pests is often required as part of maintaining crop yields, a reliance on using chemical pesticides as the main method of control is becoming increasingly difficult which has led to the increasing implementation of Integrated Pest Management (IPM) systems that reduce chemical pesticide use. IPM begins with improving plant health resulting in a crop that is less susceptible to pest damage and includes biological methods wherever possible.

## The major insecticide groups in current use

### Organophosphates

Organophosphates were first developed and used in the 1940s and 1950s, but their use increased dramatically as limits and then bans were placed on chlorinated hydrocarbons. Organophosphates have a shorter residual period and include chlorpyriphos, malathion and fenitrothion, which are principally contact insecticides [4]. The use of organophosphates became very widespread because of their rapid high efficacy and cost-effectiveness [5]. These pesticides are classified by WHO (Table 1), as moderately hazardous (Class II) and are nerve agents that inhibit cholinesterase, the enzyme that hydrolizes the neurotransmitter acetylcholine, essential for functioning of the nervous system [6]. The latter characteristic has provided a readily available test for human health in that blood cholinesterase levels could be monitored in those involved in regular application of these pesticides. Fenitrothion is used to control agricultural pests in many parts of the world and malathion has widespread use against locusts in China and was applied during the recent locust upsurge in Africa [6]. Chlorpyrifos has widespread use in many countries and for locust management in Africa [6].

**Table 1.** WHO classification of pesticides by acute mammalian toxicity.

			LD_50_ for the rat (mg/kg body weight)	
Class			Oral	Dermal
Ia	Extremely hazardous		<5	<50
Ib	Highly hazardous		5–50	50–200
II	Moderately hazardous		50–2000	200–2000
III	Slightly hazardous		Over 2000	Over 2000
U	Unlikely to present acute hazard		5000 or higher	

Organophosphates can cause mortality to natural enemies, aquatic organisms [7] and are acutely toxic to bees, so there needs to be buffer zones around bee hives, and to protect both honeybees and native bees, spraying crops that are in flower needs to be avoided [8,9]. These effects on non-target organisms have led the USA to restrict fenitrothion use to ant and cockroach baits. The European Food Safety Authority declared chlorpyrifos to be a significant concern to human health [10] leading to its use being banned in the European Union except for use against pests of tomatoes and grapevines. While there is still some production of chlorpyriphos in the EU [11], mainly for export, the EU has banned its presence in imported products and for countries that still allow the use of chlorpyrifos, this has led to export shipments being rejected due to the presence of trace amounts of this chemical. Organophosphates are becoming more generally recognized as environmental contaminants [12], which has led to limitations being placed on organophosphorus compounds by most western countries such that the future of this group of chemicals is uncertain.

### Carbamates

Carbamates are a large family of insecticides that include carbaryl, aldicarb, and bendiocarb. Like organophosphates, carbamates are cholinesterase inhibitors, but this inhibition is reversable, reducing the duration of toxicity [13]. Carbamates also tend to break down more quickly in the environment, reducing the duration of environmental side effects [13]. Most are classified as moderately hazardous (Class II). Bendiocarb poses moderate mortality risk to fish, mammals, non-target soil insects but is a high risk for pollinators and beneficial insects [5]. Bendiocarb was also classified in the WHO Hazard Class II of acute oral toxicity, Class III of acute dermal toxicity, and GHS Hazard Category 3 of acute inhalation toxicity, posing moderate overall human toxicity [5]. Some of the carbamates such as aldicarb, carbofuran and propoxur are very toxic to both mammalian and avian wildlife and have caused their deaths even when used at recommended levels [14]. Carbaryl has widespread use in the US for commercial agriculture, in forests, rangelands and in home gardens. Some carbamates including aldicarb and aminocarb have been completely banned while others such as carbofuran have been banned in the US and EU but still have widespread use in South America and Asia [15]. Due to its toxicity, methiocarb use in plant protection was withdrawn in the EU in 2020 [Commission Implementing Regulation (EU) 2019/1606].

### Phenyl pyrazoles

Phenyl pyrazoles such as fipronil have had widespread use in crop protection. Fipronil acts by binding to allosteric sites of GABA receptors and glutamate-gated chloride (GluCl) receptors of insects in that it prevents the opening of chloride ion channels and leads to an overabundance of neurons reaching action potential. Fipronil binds to the receptor sites and stays there, gradually reaching a lethal dose which means that fipronil is not as fast acting as other insecticides. However, it works at significantly lower doses and has prolonged residual activity [7,16]. Its longer residual activity means it has been used as a barrier treatment against locusts with fipronil applied in strips several hundred metres apart which minimizes the treated area, reducing environmental impact [16]. The marching behaviour of untreated locust bands between the strips means that the locusts readily encounter the treated strips within a few days [8]. There is widespread use of fipronil against pests of crops and of livestock, but while it has low acute human toxicity [5], fipronil’s broad-spectrum effects on invertebrates make it a significant threat to non-target organisms, such as aquatic arthropods and decapod crustaceans and soil arthropods essential to ecosystem functions [5,17–19]. Detrimental effects on bees have led to it no longer being used for plant protection in the EU for the past decade [6].

### Synthetic pyrethroids

Synthetic pyrethroids such as deltamethrin and lambda-cyhalothrin are commonly used for crop protection, public health and against household pests. They have a fast broad-spectrum activity with a rapid knock-down effect, but there can be recovery at sub-lethal doses. Deltamethrin and lambda-cyhalothrin act as neurotoxic insecticides by blocking sodium channels [6,20]]. They are classified as Class II in oral toxicity to humans but are highly toxic to bees [5,20]. Deltamethrin has low toxicity to aquatic organisms and terrestrial vertebrates, but lambda-cyhalothrin has high risks to aquatic arthropods and moderate risks to non-target organisms [5]. There has been some development of resistance in mosquitos [21] and bedbugs [22].

### Neonicotinoids

Neonicotinoid insecticides (NNIs) are currently the most widely used class of insecticides and have been registered in more than 100 countries, with extensive use in Asia and the Americas. NNIs are applied as foliar sprays, trunk injection, root drenching [23] or as seed treatments and are readily absorbed throughout plant vascular tissue, which results in systemic protection against insect pests [24,25]. This long-lasting systemic action leads to high control effectiveness. The imidacloprid NNI is one of the most widely used insecticides in the world being effective against sucking insects as a systemic insecticide, as a seed treatment for pest prevention, and for veterinary purposes including tick and flea control [26]. NNIs bind to nicotinic acetylcholine receptors (nAChRs) of a cell that are common in the central nervous system: high levels overstimulate and block the receptors [27] leading to paralysis and death. Insects have different receptor subunits and structures than mammals and NNIs bind much more strongly to the type of receptors found in insects so that they are very toxic to insects, while having a lower acute toxicity to mammals. However, the longer-term toxicity to mammals by NNIs is increasingly recognized [19,28,29]. Most neonicotinoids are water-soluble, which makes them break down slowly in the environment, so that they have long-lasting effects making them useful as systemic insecticides either when applied as foliar sprays or when used as seed treatments that are taken up as the plant grows, giving long-term protection from pests and diseases.

However, while their long-lasting effectiveness may be of value in pest management, it means that NNIs can have substantial effects on non-target organisms. Of particular concern is their association with deleterious effects on honeybees with laboratory studies finding that sublethal doses effect flight and navigation [30]. And field studies have shown that NNIs carried back to the hive lead to mortality of workers and even of queens [31]. In addition, effects on native bees have been underestimated in most studies [32] as native bees nest in the soil where NNIs can persist for some time especially when used as seed coatings. The high solubility of NNIs in water means they can readily enter waterways through run-off and have been detected in waterways in many countries including USA where Hladik *et al*. [33] found NNIs in 2/3 of the 48 streams tested, and in China [34]. Many aquatic organisms are quite susceptible to NNIs and effects on aquatic organisms at field dose levels have been detected [35]. There have been increasing concerns of with potential effects of NNIs on human health as NNIs have been detected in drinking water in the USA [36]. In agricultural areas of the Philippines, NNIs were in 81% of samples of people’s hair with both drinking water and foods eaten postulated as NNI sources [37], while in a study in China, NNIs were commonly detected in school-aged children [38]. The potential effects on human health and deleterious effects on a wide variety of non-target organisms have led to increasing scrutiny of the widespread use of NNIs [39].

## Reducing chemical pesticide use through IPM

The increasing restrictions on chemical pesticide use in many jurisdictions has meant that it has become increasingly important to have a ‘safer’ approach to insecticide use as part of ensuring their continued use. A critical part of safer use is having chemical pesticides as only one part of an Integrated Pest Management (IPM) system. IPM begins with improving plant health, resulting in crops that are less susceptible to pest damage. It relies on a high level of knowledge about the pest based on a program of scientific research, often conducted internationally, and includes biological methods wherever possible as a first option. While chemical pesticides are sometimes still needed, their use is often almost a last resort, leading to reduced chemical pesticide use that minimizes side effects on the environment and human health.

### IPM for rice

For rice, a great deal of research has been conducted by the International Rice Research Institute based in the Philippines, with offices in 17 countries. In recent years, high-quality rice meets the standards of the Sustainable Rice Platform (SRP). SRP methods [40] include periodic flooding of paddies (alternate wetting and drying) that reduces carbon dioxide emissions, and IPM of insects, weeds, and diseases that begins with balanced nutrient applications that make for a healthier crop that is less susceptible to pest damage. Pest numbers are suppressed by retaining plants that are hosts for natural enemies and removing plants that are alternate hosts for pests. Regular scouting is conducted as part community monitoring of potential pest outbreaks so that biological methods can be used while pest populations are at moderate levels. If other methods are not effective, chemical treatments are used and regular scouting ensures chemicals are used before pest populations reach high levels such that the resulting treatments are on a smaller scale. When pests are managed with this IPM system as part of SRP, then retailers are allowed to market their rice both locally and for export as meeting SRP standards, often at a premium price [40].

### IPM for cotton

For cotton, Australia has an area wide management (AWM) system for pests [41]. AWM is a regional approach to IPM that coordinates strategies for pest control and weed management amongst farms in a district. AWM promotes synchronized planting times to avoid continuous host availability and conserves habitats that shelter natural enemies. Continuous monitoring for pests is conducted, and data is often sent to a central authority that makes forecasts of future developments so that coordinated treatments can be conducted early as part of preventive management.

### IPM of locusts

For locusts, Uvarov [42] suggested that treatments should begin early in outbreaks to reduce the populations before they can damage crops. Early treatments of species with localized outbreak areas have often been able to prevent plagues, though with species that are widespread, plagues still occur from time to time but on a reduced scale resulting in much reduced crop damage [43]. Preventive management through World’s Best Practice [44] has been adapted in many parts of the world and relies on accurate forecasting, where regular locust surveillance data are downloaded into a Geographic information System that uses rainfall and other weather factors important in locust population dynamics to forecast future developments. Accurate forecasts are combined with regular surveillance so that outbreaks can be detected early and treated. It is generally recognized that it is extremely difficult for landholders alone to protect crops against migrating locusts, so government intervention is often necessary. While government and international organizations need to resort to widespread aerial spraying at times, they reduce the amount of chemical pesticide applied by using Ultra Low Volume techniques that allow more exact control of droplet spectrum so that pesticides are to be applied at lower doses [43,44]. Some programs further reduce chemical pesticide use by applying spray in strips for marching bands [8,45] or by treating very dense roosting swarms [44]. In addition, biopesticides are used in environmentally sensitive areas or where chemical pesticides cannot be used as part of being able to treat locusts wherever they are found [43–45]. These techniques have proven to be very effective in managing locust populations while reducing the risk to the natural environment and human health.

### IPM demonstration in corn and watermelon crops in USA

In a 4-year study in Indiana USA, where corn and watermelon crops predominated, conventional management that included prophylactic use of neonicotinoid coated seeds was compared with an IPM system where pesticides were only used when pests reached economic thresholds [46]. There was a much-reduced use of chemical pesticides as only 5% of IPM crops needed treating, leading to substantial cost savings. And IPM watermelon had a 129% increase in flower visitation by pollinators, mainly by wild bees, and a 26% higher yield.

### IPM as reduced chemical pesticide use

While the concept of IPM has been discussed for more than half a century [47], its implementation has often been limited in scope, with chemical pesticides still the most used method for pest management in many agricultural systems. However, in the face of increasing restrictions on the widespread use of chemical pesticides, as evidenced by the sustainable use of pesticides Directive of the European Union [48], IPM is becoming an essential part of sustainable agriculture. The EU legislation states that IPM must be implemented by all professional users as part of reducing their use of chemical pesticides. And as demonstrated by [46] in the Indiana study and by [44] for locust and grasshopper management, IPM properly applied can lead to substantial reductions in chemical pesticide use, significantly limiting side effects on the environment and human health. A critical part of IPM is to monitor pest populations and treat only when they reach either economic thresholds (in cropping situations) or when forecasts indicate the danger of outbreaks (as with locusts). Sustainable agriculture includes a variety of alternatives to chemical pesticides in its pest management systems [49], but there are times when chemical treatments are required as part of ensuring high crop yields. Complete banning of all chemical pesticide use is not yet possible. Sri Lanka banned the imports of fertilizer and pesticides during 2021, and this led to a disastrous decrease in yields. There was an overall 32% decrease in rice yield [50], with decreases of 53% in some areas [51], while tea production declined by 18% [52]. The overall result for Sri Lanka was much reduced export earnings and a substantial increase in food prices locally.

## The importance of registration in limiting side effects of chemical pesticides

Recognizing that chemical pesticides are important for crop protection and for maintaining crop yields, registration authorities require intensive testing of pesticides to ensure continued effective use against pest species while minimizing side effects on humans, non-target organisms and the environment. Registration promotes the proper application of chemical pesticides against target pests while minimizing side effects via careful use. Careful safe use includes consideration of potential harm during and after applications to pesticide users, bystanders and the general public. Users of pesticides and bystanders in the application process may be affected by dermal exposure, ingestion (from, for example, failure to wash hands after application), inhalation of spray, dust or gas, or dermal exposure [53]. Those applying pesticides need to wear personal protective equipment (PPE) and need training in avoiding direct contact with the pesticide. As a minimum PPE required is rubber gloves for mixing product liquid or powder with water for spraying and, for applicators and bystanders, long-sleeved shirts, long-legged trousers and working boots. The risks of some PPE not being worn because it is too expensive or uncomfortable in hot climates should be considered during the registration process. Critically important is avoidance of off-target pesticide drift, whether onto other people, or nearby crops, waterways and dwellings [53]. An occupational hazard recently identified from pesticide use concerns handling of cut flowers by florists (and potentially their customers). Unlike food products, there are no maximum residue limits (MRLs) set for pesticides in such products and a Belgian study [54] showed that florist may absorb pesticides through their skin which then show up in urine.

It is important to minimize chemical pesticide levels in food for humans, in feed for their pets and livestock, by applying at recommended doses and adhering to with-holding periods. Secondary effects on the environment must also be considered, such as birds feeding of insects on sprayed crops [55]. Furthermore, a most common deleterious effect of all chemical pesticides is on insect pollinators which can pick up substantial doses of chemical pesticides as they fly from plant to plant. For honeybees, locust control organizations like the Australian Plague Locust Commission have a buffer zone from beehives for spray applications [8]. In situations where locusts are near hives, beekeepers may be asked to move their hives, particularly where locusts are near crops and treatments are needed for crop protection. However, the effects on native bees have been underestimated in most studies [32,56,57], and as part of protecting other pollinators, the APLC avoids spraying crops when they are in flower.

As part of the registration process for a chemical pesticide, all of the safety features mentioned above form part of the registration package. Included is ecotoxicology, which may be described as ‘the study of harmful effects of toxic pollutants in ecosystems of not only the chemicals themselves but also their by-products and/or breakdown products. The objective of ecotoxicology is to obtain data for risk assessment due to the presence of such pollutants in the environment’ [27]. The hazards from insecticides and other pesticides in soil, water and waste material are recognized in several international treaties.

## First steps in assessing pesticide toxicity

Initial steps in the evaluation of a pesticide for registration include risk assessment of potential hazards arising from its use, followed by consideration of efficacy in controlling the target pest or pests. The very basics are set by the World Health Organization’s (WHO) classification of pesticide according to hazard classes [58]. This is based on mammalian toxicity and applying to hazards in application and particularly food safety (Table 1).

The first criterion for this classification scheme is the dose required for 50% mortality of test rat laboratory populations (LD_50)_) applied both orally and dermally. However, the resulting classification is adjusted according to reports of accidental severe poisoning/fatality (Ia, Ib, II classes), skin irritation (III). The risk of serious injury or suicide from self-poisoning particularly in low-income countries [59,60] may be considered by national regulators [61]. Although not an insecticide, the particular case of the non-selective/contact herbicide paraquat is worth mentioning. Currently classed II, previously III, it has been banned in several countries because of suicides and severe or fatal injury to applicators [51] while in some jurisdictions e.g. Western Australia, it has been given a high toxicity rating [62–64].

## Registration processes

The necessary registration procedures and the data required from manufacturers or agents of a pesticides for national registration processes are fully supported in the FAO International *Code of Conduct for Pesticide Management* [5]. The associated *Guidance on pesticide legislation* [65] provides the international formative framework (‘standards’) for pesticide legislation. Key points to note are:

1. An independent pesticides authority is necessary, either as a Statutory Body reporting to the Minister responsible for agriculture or an Agency reporting to Parliament. The ministry for agriculture is FAO’s preference for the ministry primary supporting this body rather than the health or environment ministries. However, this may be a source of conflict between rival ministries in some jurisdictions. Above all, it is necessary to avoid conflict of interest by ensuring that the pesticide regulator is kept separate from government officials who are responsible for advising users such as farmers, dispensing them as part of public programmes or directing their usage [66].
2. Also required is an Advisory Council to advise the authority on policy. Normally this body would comprise delegates from all the relevant ministries, the productive sectors, non-governmental bodies concerned with environmental protection and health. A contentious issue is whether the agrochemical industry should also be part of the Advisory Council, but they certainly should not be involved in the registration committee [67].
3. A registration committee reviewing the data dossiers supplied by the manufacturer or the manufacturer’s agent in the case of imports, fielded by technical experts on the use of pesticides in agriculture, horticulture livestock husbandry in the first instance. Other experts may be co-opted as necessary for e.g. public health use [5,65].
4. If the registration committee recommends approval/registration of a pesticide, they may recommend a specific category of registration depending on the non-target hazards it presents and/or whether there are restrictions on the crops to which it is applied. Typical categories are *general use*, *restricted* and *severely restricted*. The latter two categories generally require a permit or licence for the applicator to buy and use. Further details, including emergency authorizations are given in [67].
5. In a unitary jurisdiction, the committee will recommend approval of the active ingredient (a.i.) and registration of the product to the governing board of the pesticides authority and the Registrar of Pesticides.
6. In a federated or devolved jurisdiction, or a multistate economic bloc like the EU, there is the option of the ‘EU model’ for registration (Regulation (EC) 1107/2009). With this approach, the federal or central authority approves the a.i. (Chapter Two of the above Regulation) but leaves it to the EU member state (Chapter Three of the above Regulation), province or devolved nation’s authority to register products that are useful considering the pest prevalence, crops being grown, etc. This model is used in Australia [68]. The implications for this in internal trade are considered later.
7. The potentially most contentious issue in registration is whether approval of the a.i.(s) being reviewed should *balance* the risks of harm with the benefits of using it or deny approval if the risks of harm are sufficiently high irrespective of the benefits. In the EU, Regulation (EC) 1107/2009 repealed the long-standing previous Directive and adopted the precautionary principle in relation to risks of harm: Recital 10 of the Regulation states ‘Substances should only be included in plant protection products where it has been demonstrated that they present a clear benefit for plant production and they are not expected to have any harmful effect on human or animal health or any unacceptable effects on the environment’.
8. As mentioned above, the manufacturer, or the manufacturer’s agent for an imported pesticide, is responsible for submitting the required data dossiers, and for providing any supplementary information that may be requested to complete the data review. These data cover test data for mammalian toxicity, eco-toxicity, efficacy on specified crops, and residue persistence. In some cases, new field trials may be required in the country of registration when, for example, and ‘off-label’ crop is being considered or when the climatic and ecological conditions are markedly different from those considered for the ‘originator data’. Pesticide authorities in the Global South or small jurisdictions may not have the resources to repeat these field trials. To avoid the conduct of more toxicity on grounds of animal welfare, the registration committee might have recourse to accept pesticides registered under an **equivalent registration system** in another country, perhaps and preferably under a regional data sharing agreement. Alternatively, there could be a rule to accept pesticides registered in an OECD country. However, in these cases, care should be taken to determine what was actually registered, the a.i. or a product that might or might not be useful in the adopting jurisdiction. This has bearing on the meaning of ‘pesticide’ in legislation, care being taken to distinguish a.i. and ‘product’ [66], that includes not only the a.i. but adjuvants and other ingredients that might increase toxicity.
9. The related issue to data is registration of generic pesticides. With products with expired patents such as chlorantraniliprole, flubendiamide and sulfoxaflor [69], there should theoretically be no problem in registering generics, although testing of product composition might be necessary to avoid fraudulent or substandard products. However, the agrochemical industry introduced the controversial concept of ‘equivalence’ (to be distinguished from *equivalent registration system*) in an effort to limit the use of generics in favour of the branded product [66,70]. Guidance on the use of equivalence in pesticide registration is provided by [71].

## General and post-registration controls

Enforcement of post-registration controls is an essential follow-up of whatever controls are provided for in legislation. This enforcement is largely the responsibility of a pesticide inspectorate governed by the primary pesticides legislation and any general law governing access to premises or land with or without a warrant. One principal feature of these controls is licencing of all businesses involved in handling pesticides, including importation, storage and transport, sale (retail or wholesale) and application by professional services. This is to ensure that the premises are fit for purpose, that only registered pesticides are sold, and the businesses have a register of all transactions involving restricted or severely restricted categories. There could be exemptions for sale of domestic pesticides such as ‘fly sprays’ and some herbicides for home garden use.

Then there is the control of use, which is probably best implemented by observing the code of Good Agricultural Practices (GAP) which is a code jointly developed by government, agrochemicals industry and the agricultural community [72]. The code may be given force of law. Alternatively, members of a producers association or livestock association may be required to commit to observation of the code as a condition of membership, without which they might not be able to sell their products. A principal aim of GAP is to avoid pesticide residues in food in excess of the MRLs but also covered are personal protective equipment and safe disposal of unused pesticides to avoid environmental contamination. GAP as an element of food safety is a pre-requisite for Hazard and Critical Control Systems (HACCP) [73] in food processing or manufacture [72,74]. New data acquired during post-registration use will have a bearing on applications for renewal of registration according to registration protocols. It is noted that HACCP does not cover primary production, making safe pesticide use critically important for the food chain.

## Insecticide residues in food

Setting MRLs for pesticides in food is an extremely important public health measure and setting MRLs in imported food is one of the most significant non-tariff measures (NTMs) arising in trade issues. However, it must be stressed that an MRL sets the boundary of *legal* food safety and it should not be assumed that marginally exceeding a given MRL will actually cause any adverse effects. This is explained below in a description of how MRLs are set. It is important to understand that the presence of pesticides in food from plant protection products (PPPs) may arise not only from direct application to crops or stored products but also from off target contamination. For food of plant origin, sources of contamination can include wind-borne off target drift, runoff into soil and water, leaching into groundwater from agricultural use, and contamination from pesticides still being present from past uses. For food of animal origin, contamination can arise through presence in feed and fodder to which PPPs are applied, or animal exposure to contaminated soil, water or air [75].

## MRL setting

**Figure 1.**
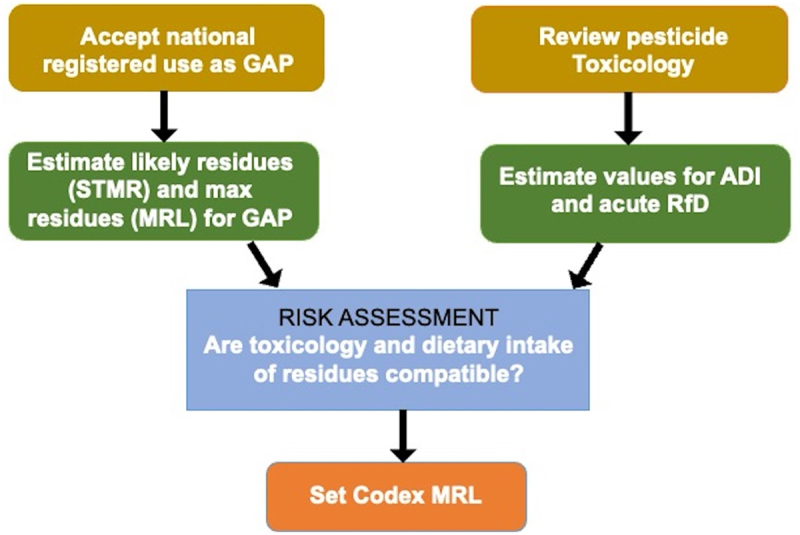
JMPR procedures for estimating pesticide MRLs. (redrawn from Yang, overview of the JMPR estimation of pesticide residues, https://www.wto.org/english/tratop_e/sps_e/wkshop_oct16_e/s2_yong_zhen_yang.pdf).

The accepted methodology adopted by the international committee of pesticide toxicologists working with Codex known as the Joint Meeting on Pesticide Residues (JMPR) is ‘working forwards’ from residue data from supervised trials of a PPP taking into account an estimate of maximum typical consumption that would present a health risk to consumers. For chronic toxicity or other harmful effects, the consumption limit is the acceptable daily intake (ADI). For acute effects, such as breathing difficulties, rash or even fatality, the consumption limit is the acute reference dose (ARfD). The ARfD is always equal to or greater than the ADI (expressed as mg/kg of body weight) (Figure 1) [76,77].

However, for ARfD, *supervised trialsmedian residue* (STMR) is used for probabilistic risk assessment, but a safety factor is applied to the MRLs obtained from trials, 10 times for adults or 100 times when the food is particularly enjoyed by children. MRLs lower than 0.01 mg/kg are no longer given, as explained below.

‘Working backwards’ starts with ADI or ARfD to derive the corresponding MRL. However, this approach is not favoured when data from supervised trials are available. Long-standing MRLs for PPPs which have been available for use without supervised trials are likely to have approval and registration reviewed by *working forwards* as above.

## What is zero MRL?

Another approach was to use the limit of detection (LOD) or limit of quantitation (LOQ): these provided the lowest possible MRL value for banned PPPs or those withdrawn from use. Strictly, the MRL for a banned substance is ‘zero’ but zero cannot be measured and analysis has to resort to LOD or LOQ. An example of LOD formerly used as ‘zero’ in the Russian Federation, MRL 0.003 mg/kg for the acaracide abamectin in tomato and grapes. At the time (2011), EU and Codex MRLs were 0.02 mg/kg [76]. However, the European Commission, followed by *Codex Alimentarius Commission* on food safety adopted 0.01 mg/kg as *effective zero* for use with a banned or withdrawn pesticide or when no MRL was available [76].

## Enforcement of food safety

To ensure food safety, it is important to have a traceable system for food products. In Australia, products are traced from ‘farm to fork’ and this is reinforced by random sampling throughout the food transport chain. Australia conducts random tests of its produce: 16,706 samples were collected during 2022–23, with only 0.5% not meeting standards as part of ensuring food safety for local consumption. Random sampling encourages self-discipline throughout the supply chain to maintain high standards so that both businesses selling products and consumers purchasing them, can be confident that food safety and the resulting benefits to human health are priorities.

Australia also samples imported products: in 2023, 19,187 analytical tests were applied to detect chemicals and contaminants at 1.1% were found to be non-compliant [78]. The EU also has a large testing program: in 2023, 132,793 samples were taken from food products, and 2.0% were found to be non-compliant [79]. This type of sampling is conducted by many importing countries which means that countries that export food must ensure food safety for their export products. It has been found that the most effective way to ensure food safety in exports is to have a high-quality food safety system for local products. However, some countries lack the resources for effective enforcement of food standards, even though there may be fully developed legislation in statute books. The result can be that food products can have high levels of chemical pesticides, and when exported a proportion have residues above MRls, leading to the rejection of the imported shipment [80]. Exporters from countries without high local safety standards, sometimes find it necessary to only purchase from farmers contracted to the business and the exporters set up their own supply chain controls from the farm to exporting facilities as part of ensuring that their exports meet international standards.

## Trade issues

Whereas a banned pesticide with an MRL of zero, ‘effective zero’ or without any recorded MRL, normally means the product’s importation will be prohibited, occasionally, some products are from particular countries may be given ‘import tolerances’ for MRLs above zero. This is a trade facilitation measure [81] and seems to reflect the issue of *process and production methods* in the exporting country according to Article 2 of the SPS Agreement. Their use in the USA is described by EPA [82]. An example for the EU are two neonicotinoids, clothianidin and thiamethoxam, for which there *were* provisional import tolerances [83], but these were removed in January 2026 and MRLs set at 0.01 mg/kg.

Developing the arguments presented above for the separation of approval of active ingredients (a.i.s) and product registration, there is a need for consistency in legislative measures for internal trade for products crossing borders in federated or devolved jurisdictions. Approved a.i.s centrally may not find any uses in one or more states or provinces. However, if there is a statutory internal or single market, products made with permitted pesticides will still be allowed on the market in a province or state in which the use is not allowed. If on the other hand, the state or province is allowed to ban a.i.s under its own legislation, the question must be asked, ‘What happens to cross-border trade from a province in which the a.i. is permitted?’

## International Convention on hazardous Products

Substantially effecting trade are the three international conventions that regulate the movement of hazardous products. *The Rotterdam Convention on the prior informed consent procedure for certain hazardous chemicals and pesticides in international trade* came into force in 2004. The Rotterdam Convention requires exporters of chemicals listed in its Annex III to receive prior-informed consent (PIC) from the **designated national authorities** of the intended importing country that is contracting partner to the Convention.

*The Stockholm Convention on Persistent Organic Pollutants* (POPs) entered into force in 2004. This Convention goes further than Rotterdam in requiring contracting parties to prohibit or severely limit the production and use of POPs listed in Annex A or Annex B with specific exceptions and only to import for the purposes of ‘environmentally sound disposal’. Importation is governed by PIC as under Rotterdam. Annex A includes detailed notes on the chemicals for which there are exceptions while Annex B covers specific restricted uses, most notably for DDT for disease vector control.

Finally, the *Basel Convention on the Control of Transboundary Movements of Hazardous Wastes and Their Disposal* is the longest standing of the three Conventions, coming into force in 1992. However, there have been major amendments, the last being in 2025 taking into account demands for stricter regulation from developing countries, alternative provisions among OECD countries and the non-signatory status of the USA. The convention originated from outcry in the Global South about the dumping of toxic waste exports from industrialized countries. The aims are:

- the reduction of hazardous waste generation and the promotion of environmentally sound management of hazardous wastes, wherever the place of disposal;
- the restriction of transboundary movements of hazardous wastes except where it is perceived to be in accordance with the principles of environmentally sound management; and
- a regulatory system applying to cases where transboundary movements are permissible.

Waste categories include A4030 Wastes from the ‘production, formulation and use of biocides and phytopharmaceuticals, including waste pesticides and herbicides which are off-specification, outdated, or unfit for their originally intended use’. PIC applies where a particular type of waste has not been prohibited.

## Conclusions

While chemical pesticides have a clear value in increasing yields through protecting agricultural products from pests and diseases, the increasing restrictions on chemical pesticide use in many jurisdictions has meant that it has become increasingly important to have a ‘safer’ approach to insecticide use as part of ensuring their continued use. Undoubtedly, since the groundbreaking publication of *Silent Spring* [1], the use of pesticides has progressively been made safer, starting with registration and regulatory action to take the most hazardous pesticides off the market, and avoiding or reducing hazards in the pesticides that remain in use. The increased understanding of both the short and long-term effects of pesticide applications has meant a pesticide must meet increasingly high standards for it to be successfully registered. As a result, registration authorities require intensive testing of pesticides, not only for their efficacy against target pests but also for their effects on humans, non-target organisms, and the environment. As part of the registration process, the pesticide must show that its clear benefits outweigh any potential harm that might occur from its use, though some jurisdictions like the EU apply the precautionary principle in that substances are not expected to have any harmful effect on human or animal health.

As part of making food safer to consume while limiting effects on the environment, the effects of chemical pesticides need to be limited to the crop they are protecting. The product must be handled safely through appropriate personal protection equipment for those mixing and applying the product and it must be applied with application equipment that meets standards that ensure that the product is applied in the proper manner and to the target only. Applicators must be trained to ensure product is applied under the appropriate environmental conditions that avoid off target drift onto adjacent areas or on to the general public. Applications must avoid effects on non-target organisms, of which native bees and honeybees are amongst the most susceptible because of their foraging behaviour. To avoid pesticide levels above MRLs in harvested products, the timing of the application must meet withholding periods prior to harvest, at a dose that will suppress the pest without overdosing. The proper, regulated use of chemical pesticides ensures crop yields so that the food we eat is safe and at a price that is affordable.

In the face of continuing scrutiny of the side effects of chemical pesticides, it is increasingly important to minimize their use through the implementation of Integrated Pest Management (IPM). IPM uses a wide variety of techniques to promote plant health and reduce pest pressure and when treatments are needed, biological alternatives are combined with the safe judicious use of chemical pesticides. The result is sustainable agriculture that includes a variety of alternatives to chemical pesticides with minimal side effects.

However, while many countries have such sustainable agricultural systems that minimize chemical pesticide use, in others sustainable agriculture faces challenges. In some countries, powerful forces encourage widespread chemical pesticide use, while others just do not have sufficient resources for either effective enforcement of legislation or implementation of IPM. In such situations, exported products, important in retaining a healthy balance of payments, may at times by rejected because of high levels of chemical pesticides. To ensure safety of both local and exported food products, there is a need for an enforcement of standards and training in sustainable agriculture that minimizes chemical pesticide use. And for all countries, the increasing understanding of the range of side effects that chemical pesticides have on both human health and the environment means that ever increasing standards will need to be met, such that the judicious use of chemical pesticides as part of IPM will be essential.
